# A framework to map cAMP signalling nanodomains: phosphodiesterase-centred phosphoproteome–interactome networks (pPINs) in cardiac myocytes

**DOI:** 10.1530/JME-26-0089

**Published:** 2026-09-23

**Authors:** Duangnapa Kovanich, Manuela Zaccolo

**Affiliations:** ^1^ Center for Vaccine Development, Institute of Molecular Biosciences, Mahidol University, Nakhon Pathom, Thailand; ^2^ Department of Physiology, Anatomy and Genetics, University of Oxford, Oxford, United Kingdom

**Keywords:** cAMP signalling nanodomains, phosphoproteome–interactome networks (pPINs), phosphodiesterases, compartmentalised signalling, integrative proteomics

## Abstract

Intracellular signalling is commonly represented as linear pathways that connect receptors to downstream effectors. While such models have been instrumental in defining signalling cascades, they fail to capture the spatial organisation that underlies signalling specificity in living cells. The cyclic adenosine monophosphate (cAMP) pathway provides a well-established example of this principle, where signalling is compartmentalised into nanometre-scale domains that generate highly localised and functionally distinct responses. However, a comprehensive framework for defining the molecular composition, spatial organisation and functional outputs of these signalling domains and how they adapt to perturbations remains lacking. Here, we discuss how integrative proteomics approaches can be used to reconstruct the subcellular cartography of compartmentalised signalling networks. By combining isoform-specific interactomics, quantitative phosphoproteomics, network analysis and spatial annotation, individual signalling platforms can be mapped within their native intracellular context. We introduce phosphoproteome–interactome networks (pPINs), a systems-level framework that integrates molecular interactions, subcellular localisation and phosphorylation responses to define phosphodiesterase (PDE)-centred signalling platforms and their associated signalling outputs. Using PDE3A isoforms as an example, we illustrate how pPINs uncover multiple spatially distinct cAMP signalling nanodomains in cardiac myocytes and reveal previously unrecognised biology, including a nuclear PDE3A2/SMAD4/HDAC-1 platform that locally constrains PKA activity and suppresses prohypertrophic gene expression. More broadly, pPINs provide a conceptual and computational framework for resolving the spatial architecture of intracellular signalling networks and establish a foundation for precision therapeutic strategies targeting discrete signalling microenvironments.

## Introduction

Intracellular signalling has traditionally been conceptualised through linear or pathway-centric models, in which stimuli transduce their signal through a sequential chain of biochemical events connecting receptors to downstream effectors. A typical example of such a representation is provided by pathway maps in the KEGG database (1). These models have been instrumental in defining core signalling components and clarifying cause–effect relationships within biochemical signalling cascades. However, they provide an inherently simplified representation of signalling processes that, in living cells, are highly multidimensional. Linear pathway descriptions treat protein components as single entities operating in a homogeneous, largely context-independent intracellular environment and assume pathway modulation occurs via unrestricted diffusion and ‘bulk changes’ in the level of the relevant second messenger. As such, these models fail to capture the spatial, temporal and organisational constraints that fundamentally govern how signals are generated, transmitted and interpreted.

In eukaryotic cells, signalling unfolds within a highly structured intracellular landscape. Subcellular compartments, membrane systems, cytoskeletal networks and macromolecular assemblies create spatially confined environments in which biochemical reactions are locally regulated. Eukaryotic cells repeatedly reuse a limited set of second messengers, kinases, phosphatases and other molecular components across numerous pathways. Subcellular compartmentalisation of these components enables cells to process biochemical information locally, ensuring that signalling outputs are precise, non-diffuse and stimulus specific, even when multiple pathways that share the same signalling elements are activated concurrently. Thus, the same second messenger or kinase can produce divergent functional outputs due to differences in localisation, temporal dynamics and the molecular architecture they operate within, rather than changes in their intrinsic biochemical properties or overall abundance.

Linear representations of pathways obscure these key contextual determinants, offer limited insight into differential responses across subcellular regions and constrain our understanding of physiological states and disease conditions. Instead, this biological reality can be captured by compiling a detailed annotation of signalling components according to their subcellular organisation to provide a subcellular cartography of signalling networks. Within this framework, signalling is conceptualised as an interconnected system of spatially organised molecular assemblies, where the relative positioning of proteins, their incorporation into localised complexes and their dynamic reorganisation over time are central determinants of information flow and signal decoding.

Importantly, a subcellular map of signalling networks can reveal organisational principles that are largely concealed in linear models, including discrete signalling hotspots, compartmentalised kinase and phosphatase activity and domain-specific pathway cross-talk. Resolving signalling at this level exposes critical control points where enzymatic activity, protein–protein interactions and localised post-translational modifications converge to generate highly specific functional outputs. These control points underpin cellular adaptive responses and pathological remodelling and can become the target for precision therapeutic interventions.

## Compartmentalisation in cAMP signalling

The 3′–5′-cyclic adenosine monophosphate (cAMP)-protein kinase A (PKA) pathway provides a relatively well-characterised example of compartmentalised signalling. Modulation of cAMP is triggered by a large variety of hormones, neurotransmitters and other stimuli that activate Gs or Gi G protein-coupled receptors (GPCRs). Decades of work have established that the specificity of GPCR–cAMP signalling in transducing hormone-specific inputs is not dictated by global changes in bulk intracellular cAMP levels but instead by spatially confined cAMP gradients generated within nanometre-scale subcellular domains (2, 3, 4, 5, 6, 7, 8, 9, 10, 11) (cAMP nanodomains). These gradients emerge from the coordinated activity of adenylyl cyclases (ACs), which synthesise cAMP, and phosphodiesterases (PDEs) (12), which degrade it. Together, these enzymes couple cAMP production and breakdown to the localised regulation of cAMP effectors and, in particular, PKA, which is tethered in close proximity to defined phosphorylation targets through interactions with A-kinase anchoring proteins (AKAPs) (13) (Fig. 1). In these settings, cAMP nanodomains represent discrete signalling territories in which second-messenger dynamics are uniquely regulated and tightly coordinated, distinctly affecting kinase activation and local substrate phosphorylation. Importantly, the presence of a resident PDE within a cAMP nanodomain should not be interpreted as maintaining the compartment in a constitutively inactive state. Rather, resident PDEs act as local cAMP sinks by continuously hydrolysing cAMP, thereby shaping the local balance between cAMP synthesis and degradation, which determines whether cAMP accumulation reaches the threshold for PKA activation (14). Accordingly, the activation state of an individual nanodomain depends on this local balance. Under different physiological conditions, including activation of distinct GPCR populations or changes in receptor stimulation that alter this balance, individual nanodomains can transition between inactive and active signalling states, thereby generating receptor-specific patterns of localised cAMP signalling (Fig. 1).

**Figure 1:**
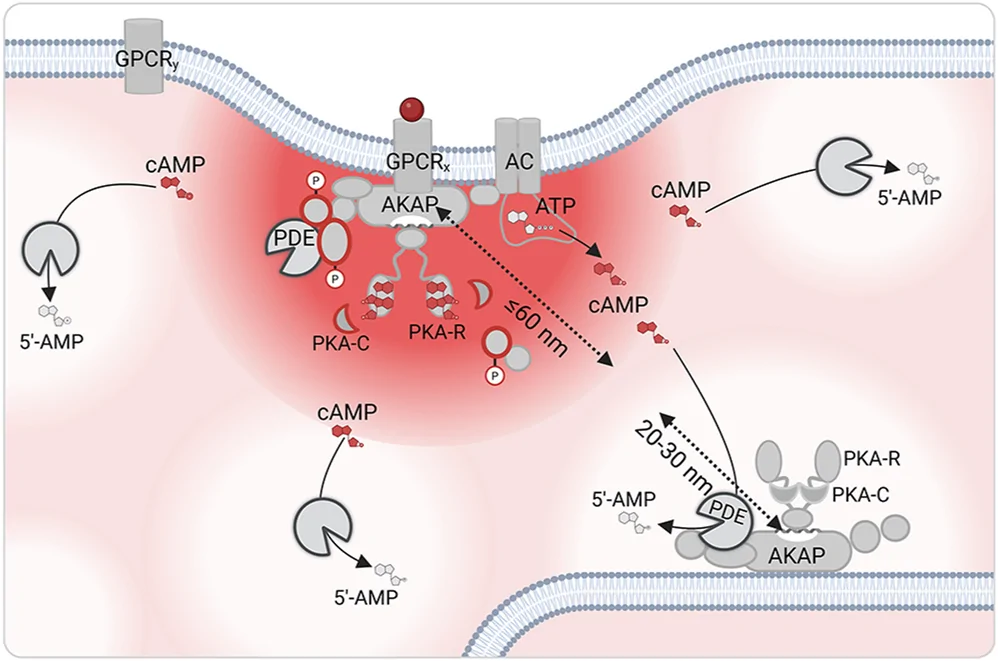
Spatially confined cAMP nanodomains control localised signalling platforms organised by PKA scaffolding proteins. Schematic illustration of receptor-associated cAMP signalling nanodomains. Two distinct G protein–coupled receptors (GPCRs), termed GPCRx and GPCRy, are shown at the plasma membrane. Binding of a ligand to GPCRx activates Gs signalling and stimulates adenylyl cyclase (AC), generating a localised pool of cAMP. Within the active GPCRx-associated compartment, an A-kinase anchoring protein (AKAP) tethers protein kinase A (PKA) in proximity to defined substrates. cAMP compartmentalisation results from cAMP buffering, which slows down its diffusion, and the activity of the resident phosphodiesterase (PDE), which locally hydrolyses cAMP. The schematic shows a receptor-associated nanodomain with a size of up to approximately 60 nm that results from such organisation (7). This spatial range is comparable to the distance over which AKAP-anchored PKA regulatory (PKA-R) subunits can extend (15, 16). The properties of the PDE associated with this domain are such that, upon receptor activation, elevation of cAMP is sufficient to activate the catalytic PKA subunits (PKA-C), which in turn phosphorylates target proteins located within the active nanodomain (encircled P symbols). Also shown is an intracellular cAMP nanodomain in which a resident PDE acts as a local cAMP sink, maintaining local cAMP below the threshold required for PKA activation during the illustrated GPCRx stimulation, in this case within a radius of approximately 20–30 nm. The size of the domain and the level of cAMP achieved within it depend on the specific hydrolytic properties of the associated PDE(s). Activation of GPCRy is expected to trigger a different pattern of subcellular cAMP nanodomains. Created with BioRender.com.

cAMP nanodomain signalling has been most extensively characterised in cardiomyocytes, establishing these cells as a paradigm for compartmentalised signalling (3, 6, 9, 11, 17, 18). As most other cells, cardiac myocytes display a highly complex signalling landscape in which diverse hormonal and neurohumoral inputs use cAMP to transmit a myriad of different signals while maintaining specificity of response with precision. These cells express a broad repertoire of Gs/iPCRs, multiple AC isoforms, diverse cAMP effectors, including multiple PKA isoforms, an extensive array of AKAPs and several families of cAMP-degrading PDEs, each comprising multiple isoforms (10). Together, this molecular diversity gives rise to numerous spatially and functionally distinct cAMP/PKA signalling platforms distributed across the cell, creating a structured signalling environment in which cAMP dynamics are locally interpreted and translated into specific functional outcomes.

For example, early studies in isolated perfused hearts demonstrated that isoproterenol, a β-adrenergic receptor agonist that mimics sympathetic stimulation, and prostaglandin E1 (PGE1), which signals through PGE receptors, induce comparable global elevations in cellular cAMP and PKA activity but result in markedly different functional outcomes (19, 20, 21). While isoproterenol stimulates the phosphorylation of key PKA substrates involved in cardiac contraction, including troponin I and phosphorylase kinase, PGE1 does not (19, 20, 21). These findings provided early evidence that cAMP signals are interpreted locally through selective activation of spatially restricted subsets of PKA, rather than by uniform signal propagation and indiscriminate activation of PKA across the cell. Subsequent studies in intact, living cardiac myocytes using real-time imaging and genetically encoded fluorescent probes for cAMP directly demonstrated compartmentalised cAMP signalling (2). These experiments revealed that cAMP elevations in response to different stimuli are not homogeneous throughout the cytosol but instead form spatially restricted, highly localised gradients (2). Moreover, the subcellular distribution and dynamics of these signals are hormone-specific, generating distinct patterns of cAMP accumulation that align with the selective activation of downstream effectors within defined nanodomains (2). Notably, the nanodomains within which cAMP concentrations differ from the surrounding cellular environment can be extremely small, in some cases spanning only a few tens of nanometres (2, 7, 8). This remarkable spatial confinement enables highly selective modulation of effectors contained within the nanodomain while preventing widespread diffusion of the signal, thereby preserving the specificity of hormone-dependent responses.

Although compartmentalisation of the cAMP–PKA pathway in cardiac myocytes is firmly established, a comprehensive map of cAMP signalling nanodomains has yet to emerge. To date, only a limited number of these domains have been characterised with respect to their subcellular localisation, molecular composition and signalling dynamics, leaving much of the cAMP signalling landscape unexplored and many cAMP/PKA-dependent functional effects without clear spatial attribution (17, 18, 22, 23, 24, 25, 26, 27, 28). Current knowledge derives largely from targeted studies employing genetically encoded cAMP sensors directed to predefined subcellular locations, typically at sites associated with well-established PKA targets involved in excitation–contraction coupling (6, 18, 27, 28, 29, 30). While these studies have yielded important mechanistic insights (6, 18, 30), their reliance on prior knowledge inherently restricts the identification of previously unrecognised signalling compartments. Consequently, the number, spatial organisation and functional interrelationships of cAMP nanodomains in cardiomyocytes remain largely undefined, representing a major gap in our understanding of cardiac signal transduction.

## Network-based and integrative proteomics approaches

Protein–protein interaction (PPI) networks (interactomes) have long provided a systems-level framework for organising complex signalling information. In these networks, proteins are represented as nodes, while their physical or functional associations are represented as edges. Interactome maps derived from experimental interaction data, curated literature and computational predictions have been widely used to identify protein assemblies, define functional modules and contextualise signalling pathways at a global scale (31). However, most current PPI resources and pathway frameworks, including STRING (32) and KEGG-derived maps (1), treat proteins as context-independent entities. Each protein is represented as a single node, ignoring the diversity resulting from isoforms and other proteoforms (e.g. post-translationally modified) and the fact that the same protein can localise to multiple subcellular compartments simultaneously, where it engages distinct interaction partners within unique molecular environments, resulting in context-specific functional outcomes.

By collapsing parallel, compartment-specific interaction networks into a single, unified representation, conventional interactome frameworks obscure the spatial organisation that underpins signalling specificity. Overcoming this limitation requires strategies that preserve the spatial context of protein interactions rather than averaging them across the cell.

Within this conceptual framework, PDEs provide a tractable entry point to resolve spatially organised cAMP signalling networks. PDE isoforms localise to discrete subcellular regions, where they sculpt cAMP gradients within a restricted radius and are embedded in multiprotein assemblies comprising scaffold proteins, kinases, phosphatases and other structural and/or functional components (17, 25, 26, 29, 33, 34). As PDEs define the boundaries of cAMP nanodomains and thereby determine where PKA is activated and where downstream phosphorylation occurs, individual PDE isoforms can serve as molecular signposts that identify functionally distinct signalling compartments. This conceptual framework enables PDE inhibition to be used as a discovery tool: disrupting PDE activity perturbs local cAMP nanodomains and reveals signalling responses indicative of previously uncharacterised nanodomains (17, 35, 36).

Inhibition of a PDE at a certain location is expected to locally elevate cAMP, leading to confined activation of PKA and phosphorylation of nearby substrates. Quantitative phosphoproteomics can then capture these phosphorylation events, enabling the identification of stimulus-dependent signalling changes and the inference of their subcellular location based on the identity and context of the regulated proteins (17, 35, 36). A major limitation of this approach is the lack of PDE isoform-selective pharmacological inhibitors. Because available compounds inhibit entire PDE families rather than individual isoforms, they simultaneously perturb multiple nanodomains across the cell. Consequently, the resulting phosphorylation responses cannot be readily linked to the activity of a single localised PDE or the specific nanodomain it governs.

To circumvent this limitation, a recent study employed an integrative proteomics approach to map cAMP nanodomains associated with individual PDE isoforms. Building on the premise that distinct PDE isoforms define discrete signalling compartments, phosphoproteomic profiling of PDE family-dependent changes in phosphorylation was combined with PDE isoform-specific interaction proteomics (17). The study aimed to identify the cAMP nanodomain landscape generated in response to *β*-adrenergic stimulation in cardiac myocytes. The phosphorylation changes induced by selective inhibition of PDE families in isoproterenol-treated cardiac myocytes were mapped onto protein interaction networks centred on individual PDE isoforms. These networks can be constructed by integrating experimentally determined PDE isoform interactomes with curated protein–protein interaction datasets (e.g. STRING) and reconstructed using network analysis tools such as Cytoscape. This integrative framework enables the deconvolution of spatially confined signalling platforms and the assignment of downstream phosphorylation events to specific cAMP nanodomains with a defined subcellular location (10, 17).

## Phosphodiesterase phosphoproteome–interactome networks (pPINs)

The integrative strategy described above provides a means to assign phosphorylation events to discrete cAMP nanodomains and relate these outputs to PDE isoform-centred signalling platforms (Fig. 2). To formalise this organisation, we define PDE phosphoproteome–interactome networks (pPINs) as spatially resolved maps of PDE isoform-centred signalling platforms that integrate three core features of compartmentalised signalling: subcellular localisation, interaction architecture and associated phosphorylation responses. In this framework, individual signalling platforms function as hubs that constrain kinase activity and specify downstream phosphotargets, thereby providing experimentally tractable representations of compartmentalised signalling.

**Figure 2:**
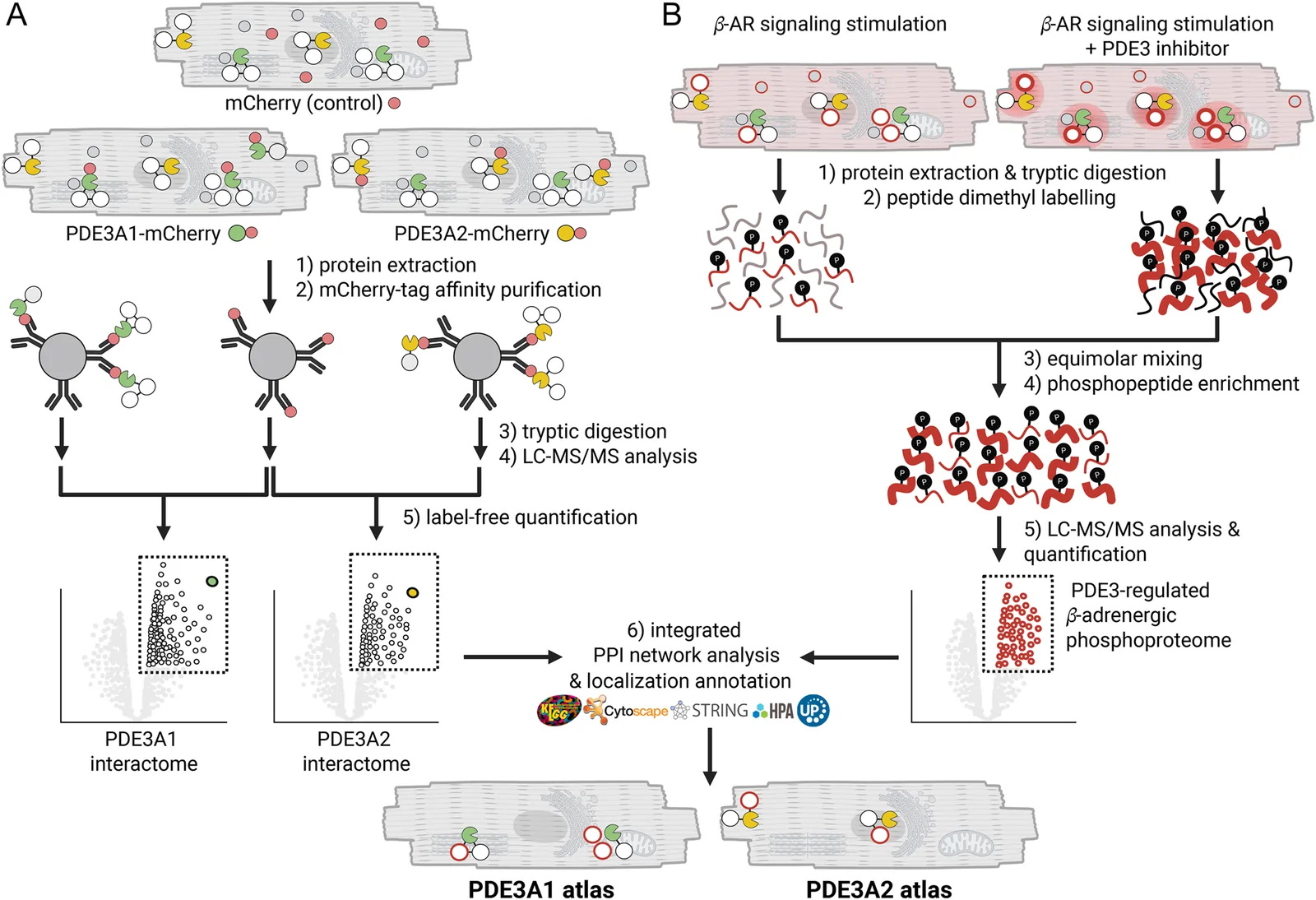
Integrated proteomics approach for the definition of PDE3A pPINs. (A) Isoform-specific interaction proteomics is performed in neonatal cardiomyocytes expressing mCherry-tagged PDE3A1 or PDE3A2, together with an mCherry control, followed by mCherry-based affinity purification–mass spectrometry to define the molecular assemblies associated with each PDE isoform. mCherry is represented by red circles, whereas PDE3A1 and PDE3A2 are represented by green and yellow Pac-Man symbols, respectively. Because protein overexpression can generate non-physiological interactions through protein mislocalisation and ectopic complex formation, interaction datasets may contain non-physiological PDE3A-associated assemblies. In the schematic, physiologically relevant interactors are represented as white circles with black borders, whereas potential non-physiological interactors are represented as grey circles with black borders. (B) Quantitative phosphoproteomics based on dimethyl labelling and phosphopeptide enrichment is carried out to capture phosphorylation changes induced by pharmacological inhibition of PDE3 activity (cilostamide) during β-adrenergic stimulation, reflecting localised cAMP/PKA signalling outputs. In cells subjected to β-adrenergic stimulation, phosphotargets are represented as white circles with red borders, whereas increased phosphorylation following PDE3 inhibition is represented by thicker red borders. Light dimethyl-labelled peptides and phosphopeptides originating from cells subjected to β-adrenergic stimulation are represented using grey and red lines, respectively. Heavy dimethyl-labelled peptides originating from cells subjected to β-adrenergic stimulation and PDE3 inhibition are represented by black lines. Increased phosphopeptide abundance following PDE3 inhibition is indicated by thicker red lines. Phosphorylation changes are mapped onto interaction-defined protein assemblies and annotated using subcellular localisation information to assign signalling networks to discrete intracellular locations. Integration of interactomic data with phosphoproteomic data enables prioritisation of interaction-defined assemblies associated with coordinated signalling responses, thereby minimising the contribution of false-positive and non-physiological interactions to the reconstructed signalling networks. This integrative framework enables the reconstruction of spatially organised PDE-centred cAMP signalling platforms associated with individual PDE isoforms and their associated phosphorylation responses, linking molecular composition, localisation and downstream phosphorylation responses across the intracellular landscape. Created with BioRender.com.

A defining feature of the PDE-pPINs is that they emerge from the integration of two orthogonal data types. Isoform-specific interactomes define the structural composition and spatial neighbourhood of the signalling platforms, while phosphoproteomic profiling captures their functional output in response to defined perturbations. Mapping phosphorylation events onto interaction-defined networks enables internal cross-validation, linking physical connectivity with coordinated signalling responses. In this way, PDE-pPINs bridge molecular organisation and function, providing a framework to interpret signalling specificity in spatially organised systems.

We applied this framework to resolve cAMP signalling platforms regulated by PDE3A isoforms, the predominant phosphodiesterases in human myocardium and a clinically relevant target in heart failure (17). Although PDE3 inhibitors produce beneficial acute inotropic effects (37), their long-term use is associated with adverse outcomes (38, 39). The fact that both protective and detrimental roles have been reported for members of the PDE3A subfamily in the heart (40, 41, 42, 43, 44) is consistent with individual PDE3A isoforms controlling distinct cAMP nanodomains with divergent functional consequences.

Analysis of PDE3A1 and PDE3A2 revealed that each isoform is embedded within multiple, spatially distinct pPINs corresponding to separate cAMP signalling platforms (10, 17). Notably, PDE3A2 defines a previously unrecognised nuclear signalling platform associated with a SMAD4–HDAC-1 complex, where it locally constrains cAMP/PKA activity to maintain repression of prohypertrophic gene expression during physiological β-adrenergic stimulation (Fig. 3A, B, C, D), thereby exerting a cardioprotective effect. Inhibition of the PDE3A2 resident within this signalling platform through its displacement using a catalytically inactive PDE3A2 mutant leads to enhanced HDAC-1 phosphorylation and activation of hypertrophic transcriptional programmes. In contrast, PDE3A1 associates with pPINs distributed across distinct subcellular compartments (Fig. 3A). Replacement of active PDE3A1 within these networks by a catalytically inactive mutant attenuates β-adrenergic-induced hypertrophy, providing direct evidence that PDE3A isoform-defined signalling platforms mediate opposing functional outcomes (17).

**Figure 3:**
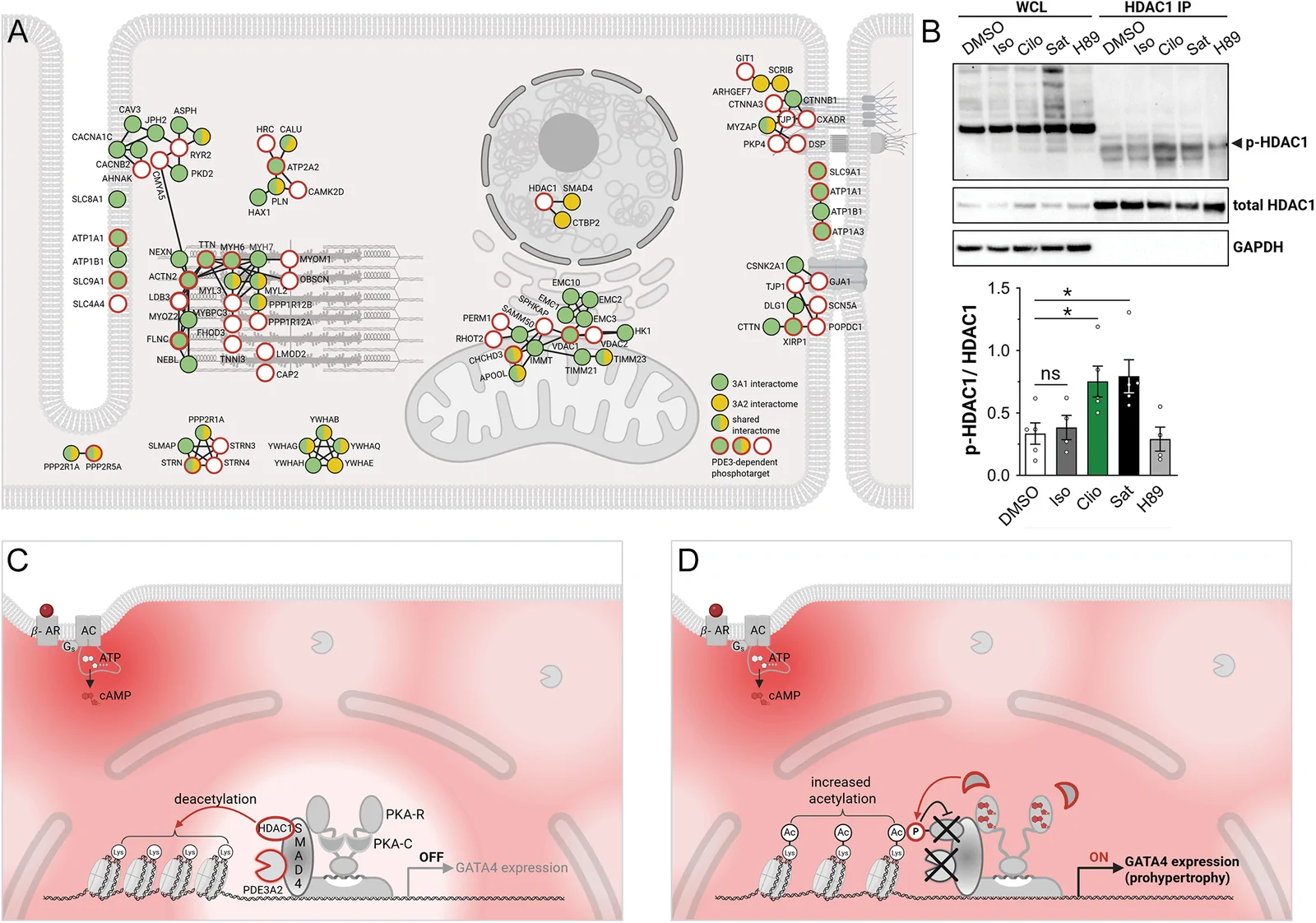
Cardiac PDE3A pPINs reveal novel biology. Integrative proteomics-based reconstruction of PDE3A-associated cAMP signalling platforms identifies a previously unrecognised nuclear PDE3A2/SMAD4/HDAC-1 signalling platform that regulates hypertrophic gene expression. (A) Subcellular localisation of a selection of PDE3A1- and PDE3A2-associated cAMP signalling platforms revealed by integrative proteomics and pPIN analysis. The spatial map is reconstructed using the PDE3A1- and PDE3A2-interactome and PDE3 inhibitor cilostamide-dependent phosphoproteome datasets reported in our previous study (17). Green and yellow nodes represent proteins identified in the PDE3A1 and PDE3A2 interactomes, respectively, while bicoloured green-yellow nodes indicate proteins shared between the two interactomes. Proteins are labelled using their corresponding gene symbols. Nodes outlined in red denote PDE3-regulated phosphotargets. Edges represent experimentally supported protein–protein interactions or associations, whereas spatial proximity of nodes reflects localisation within the same subcellular microenvironment and potential functional association. Shown are selected PDE3-associated cAMP signalling platforms with established roles in cAMP and/or β-adrenergic signalling, localised to distinct subcellular regions including the dyad, sarcomere, intercalated disc (ICD), mitochondria–endoplasmic reticulum contact sites (MERCs) and nucleus. (B) PDE3A2 restricts local cAMP accumulation and thereby regulates PKA-dependent phosphorylation of HDAC-1. Inhibition of PDE3A results in local elevation of cAMP and HDAC-1 phosphorylation. Notably, when the PDE3A2 enzyme is active, elevation of cAMP through β-adrenergic receptor stimulation at the plasmalemma does not affect HDAC-1 phosphorylation (adapted from (17)). (C) The panel illustrates the response in the presence of active nuclear PDE3A2. β-adrenergic stimulation elevates cAMP globally. However, active PDE3A2 localised to the SMAD4/HDAC-1 complex maintains local cAMP concentrations below the threshold required for PKA activation, thereby preserving HDAC-1 deacetylase activity and repressing prohypertrophic gene expression. (D) Inhibition or displacement of PDE3A2 increases local cAMP concentrations within the PDE3A2/SMAD4/HDAC-1 cAMP signalling platform, leading to PKA activation and HDAC-1 phosphorylation. This reduces HDAC-1 deacetylase activity, resulting in enhanced transcription of prohypertrophic genes and cardiac myocyte hypertrophy. ISO, isoproterenol; Cilo, cilostamide; Sat, saturating cAMP stimulus (IBMX and forskolin); H89, PKA inhibitor. Created with BioRender.com.

Thus, the pPIN framework delineates a spatial map of PDE isoform-associated signalling platforms and their molecular composition. This map provides a foundation for generating and testing hypotheses that link individual nanodomains to defined functional outcomes, offering a path to dissect how spatially organised cAMP signalling contributes to cardiac physiology and disease.

Importantly, this framework is generalisable. The same strategy can be applied to other signalling systems in which isoforms differ in localisation and regulatory context and where perturbation of pathway activity can be coupled to phosphoproteomic readouts. In such settings, integration of isoform-specific interactomes with phosphoproteomic responses enables reconstruction of spatially defined signalling platforms and their functional outputs.

## Future directions

While the cardiac PDE pPINs illustrated here are an example of how this approach can uncover the connectivity of signalling networks and provide a framework for hypothesis-driven interrogation, work done so far provides static snapshots captured under a fixed set of conditions. Moving from descriptive maps to predictive models of signalling will require understanding how these networks dynamically adapt and rewire in response to perturbations.

Although the pPIN framework provides a powerful strategy for reconstructing PDE-centred cAMP signalling platforms, several limitations should be recognised. First, the current workflow relies heavily on mass spectrometry-based interactomics and phosphoproteomics, which incompletely sample the proteome. Low-abundance signalling proteins, transient interactions and highly hydrophobic membrane proteins, including GPCRs and ion channels, remain challenging to detect and therefore may be underrepresented in reconstructed signalling platforms. Second, the functional output of the current framework is inferred primarily from phosphorylation changes and therefore captures phosphorylation-dependent signalling responses downstream of cAMP signalling. Consequently, cAMP-dependent signalling processes that are not primarily mediated through phosphorylation, such as HCN channel gating, will require complementary functional approaches for their direct investigation.

Emerging spatial proteomics approaches can help address several of these limitations by providing a critical orthogonal layer of information in the form of quantitative, cell state-specific maps of protein localisation. Fractionation-based strategies, such as Localisation of Organelle Proteins using Data-independent Acquisition (DIA-LOP) and dynamic organellar mapping (45, 46), establish proteome-wide spatial references that can be integrated with interactome and phosphoproteomic datasets to constrain the localisation of signalling platforms and improve the resolution of pPINs. Importantly, integration with total proteome measurements enables discrimination between true protein relocalisation events and changes driven primarily by altered protein abundance.

Equally critical is the temporal dimension, as signalling responses often involve rapid relocalisation, transient complex assembly and stimulus-dependent redistribution of proteins. Time-resolved spatial proteomics and proximity labelling approaches, such as BioID, TurboID and APEX (47, 48, 49, 50), enable dynamic capture of these processes *in situ*, improving detection of low-abundance proteins, transient interactions and proteins that are difficult to recover using conventional affinity purification–mass spectrometry. These approaches reveal how signalling platforms assemble, reorganise and disassemble over time, thereby refining the molecular composition and spatial organisation of pPINs. Together, spatially and temporally resolved proteomics approaches make it possible to reconstruct not only the architecture of signalling platforms and networks but also their dynamic behaviour across physiological states and perturbations. Future development of pPINs through integration of proteomic datasets with complementary functional data from genetically encoded cAMP sensors (6) and other functional approaches will broaden the framework to encompass both phosphorylation-dependent and phosphorylation-independent aspects of cAMP signalling.

Realising the full potential of these datasets will require integration with computational frameworks capable of inferring causal relationships and modelling network rewiring. Network-based causal inference (51) and multi-omics integration approaches (52) can reconstruct context-specific signalling flows, while machine learning models enable prioritisation of functionally relevant subnetworks and prediction of perturbation responses (53). In parallel, probabilistic and AI-assisted methods can refine subcellular localisation assignments, particularly for multi-localised proteins, by integrating spatial proteomics, interaction data and literature-derived knowledge, thereby improving the fidelity of spatially resolved signalling networks.

By integrating molecular organisation, spatial context, temporal dynamics and functional output within a unified and computationally tractable framework, pPINs offer a powerful approach for understanding how the nanoscale organisation of biochemical reactions within subcellular domains gives rise to physiological function and contributes to disease. More broadly, they support a view of intracellular signalling as a context-dependent information-processing system, in which signalling outcomes are determined not only by pathway composition but also by the spatial and molecular environments in which they occur. Ultimately, this framework may pave the way for a new generation of precision therapeutics that target signalling with subcellular specificity (54), improving efficacy while minimising unintended effects.

## Declaration of interest

The authors declare that there is no conflict of interest that could be perceived as prejudicing the impartiality of this work.

## Funding

This work was supported by the British Heart Foundation (RG/12/3/29423, RG/17/6/32944, PG/23/11321 and PG/21/10611), the Oxford BHF Centre of Research Excellence (RE/08/004 and RE/18/3/34214) and the Oxford NIHR Biomedical Research Centre.
